# Chaotic synchronization of two optical cavity modes in optomechanical systems

**DOI:** 10.1038/s41598-019-51559-1

**Published:** 2019-11-01

**Authors:** Nan Yang, Adam Miranowicz, Yong-Chun Liu, Keyu Xia, Franco Nori

**Affiliations:** 1 National Laboratory of Solid State Microstructures, College of Engineering and Applied Sciences, Nanjing University, Nanjing, 210093 China; 2 Theoretical Quantum Physics Laboratory, RIKEN Cluster for Pioneering Research, Wako-shi, Saitama, 351-0198 Japan; 3 Faculty of Physics, Adam Mickiewicz University, Poznan, 61-614 Poland; 4 State Key Laboratory of Low-Dimensional Quantum Physics, Department of Physics, Frontier Science Center for Quantum Information, Collaborative Innovation Center of Quantum Matter, Tsinghua University, 100084 Beijing, China; 5 Collaborative Innovation Center of Advanced Microstructures, Nanjing, 210093 China; 6 Physics Department, The University of Michigan, Ann Arbor, Michigan, 48109-1040 USA

**Keywords:** Optics and photonics, Physics

## Abstract

The synchronization of the motion of microresonators has attracted considerable attention. In previous studies, the microresonators for synchronization were studied mostly in the *linear* regime. While the important problem of synchronizing *nonlinear* microresonators was rarely explored. Here we present theoretical methods to synchronize the motions of chaotic optical cavity modes in an optomechanical system, where one of the optical modes is strongly driven into chaotic motion and transfers chaos to other weakly driven optical modes via a common mechanical resonator. This mechanical mode works as a common force acting on each optical mode, which, thus, enables the synchronization of states. We find that complete synchronization can be achieved in two identical chaotic cavity modes. For two arbitrary nonidentical chaotic cavity modes, phase synchronization can also be achieved in the strong-coupling small-detuning regime.

## Introduction

The synchronization of oscillators is a universal concept in nonlinear sciences^1,2^. It has been observed in both nature^2^ and social activities^1–3^, and also promises important applications in engineering^1,2,4–8^. Since its discovery in pendulum systems by Huygens in the 17th century^9^, synchronization has been observed in various fields including bursting neurons^10^, fireflies^11^, and chemical reactions^12^. Although these systems operate in very different size scales, the mechanism behind synchronization can be understood as follows: oscillators under weak interaction adjust their rhythms to keep their motions consistent. The synchronization of oscillators has also been studied in relation to information processing^7^, communications^5^, and high-precision clocks^8^.

Optomechanical resonators^13–31^ with high-quality factors and strong nonlinearities have attracted considerable attention in various fields due to their promising applications. The synchronization of optomechanical systems is an important topic in optomechanics^32–42^. It has been demonstrated that an optomechanical system, with strong nonlinear light-matter interaction^43–46^, can support quite different types of motion, i.e., periodic^14^, quasi-periodic^23^, and chaotic^21–23,27–29^. However, the majority of previous works concentrated on the synchronization of *periodic* oscillations. The synchronization^47^ of two *chaotic* optomechanical systems is still a very challenging task. The main problem is how to design an experimentally accessible setup, which implements existing mathematical schemes of chaotic synchronization. For instance, chaotic synchronization fails in two optomechanical resonators, when they are mediated by optical fields. Therefore, a model enabling the chaotic synchronization in optomechanical systems is desirable.

In this paper, we propose methods for the synchronization of two optical modes in an optomechanical system with *chaotic* dynamics rather than with *periodic* motion. These methods are based on a configuration in which a strongly driven and one (or more) weakly driven cavity modes are coupled to a mechanical resonator. Chaos is generated from the nonlinear optomechanical coupling^21–23,27–29^ between the strongly driven cavity mode and the mechanical mode, and transferred to the other weakly driven optical-cavity modes via the mechanical motion^23^. The mechanical resonator subsequently works as a common force acting on every cavity modes. In this configuration, we find that complete synchronization is achievable in two identical weakly driven cavity modes, and phase synchronization can be realized in two arbitrary different cavity modes in the weak-detuning strong-coupling regime.

The complete synchronization studied in this paper can be understood in the framework of the active-passive decomposition (APD) model^48–50^. In the APD model, a chaotic system is decomposed into two parts: an autonomous subsystem and a *non*-autonomous subsystem (system 1). An identical non-autonomous subsystem is called system 2, and driven by the same autonomous subsystem as system 1. Complete synchronization between chaotic systems 1 and 2 can be achieved if their synchronization error is uniformly asymptotically stable at the zero point defined right below Eq. (4). In optomechanical setups, the mechanical resonator corresponds to the autonomous subsystem, and the two chaotic weakly driven optical modes are the non-autonomous subsystems to be synchronized. By constructing a strict Lyapunov function, we show that complete synchronization can be achieved and is stable to mechanical input and initial conditions. Moreover, numerical simulations, given in this paper also coincide with this analytical result.

For two *non-identical* chaotic optical modes, the hidden synchronization, known as phase synchronization, can also be observed in the systems 1 and 2. The configuration used here is the same as in complete synchronization. The mechanical resonator acting on the frequencies of each optical resonators, modulates their rotations in the same way. Especially, in the strong-coupling small-detuning regime, the rotations of the cavity modes are dominated by the frequency shifts brought by the same mechanical resonator. Here, we use unwrapped phases to measure the rotations of the optical resonators. Thus, although the two optical modes are in different chaotic motions, their unwrapped phases can be locked at a fixed ratio.

We propose two optomechanical setups for either complete synchronization (A1 and B1) or phase synchronization (A2 and B2) of chaotic optical modes. In both setups A1 and A2, all the optical modes are integrated into a single optomechanical system and coupled via the same mechanical resonator. While in setups B1 and B2, the optical modes are coupled via different connected mechanical modes, which thus, have more potential applications as the optical modes to be synchronized are distributed in difference devices. In the strong mechanical coupling regime, setups B1 and B2 can be reduced to setups A1 and A2, respectively.

This paper is organized as follows: In Secs. II and III, we present the corresponding setups for both complete and phase synchronization in an optomechanical system. The numerical results for these two types of synchronization are shown and compared in Sec. IV. In Sec. V we summarize our work and discuss some potential applications.

## Complete Synchronization

In this section, we propose two optomechanical setups (A1 and B1) for the synchronization of two chaotic identical optical modes. These setups enable the following processes: (1) the mechanical mode transfers chaos, which is generated by a strongly driven optical mode, to other weakly driven modes, (2) it acts as a common force to synchronize each optical mode to a consistent state. When the weakly driven optical modes are identical, this configuration leads to a complete synchronization of the modes, which can be understood by the already defined APD model. Below we first introduce the concepts of complete synchronization and the APD model.

In general, complete synchronization refers to the identity among the phase-space orbits of chaotic systems. Let us consider two chaotic systems

1 $${\dot{{\bf{y}}}}_{1}={\bf{f}}({{\bf{y}}}_{1}),\,{\dot{{\bf{y}}}}_{2}={\bf{f}}({{\bf{y}}}_{2}),$$

where **y**_1_ and **y**_2_ are *N*-dimensional variables governed by the function **f**: ${R}^{N}\to {R}^{N}$. We define the difference between the phase-space orbits of two chaotic systems as the synchronization error ${\bf{e}}(t)$, where ${\bf{e}}(t)={{\bf{y}}}_{1}(t)-{{\bf{y}}}_{2}(t)$. Two chaotic systems are called completely synchronized if and only if their synchronization error ${\bf{e}}(t)$ vanishes in the evolution long time limit^51^, i.e.,

2 $$\mathop{\mathrm{lim}}\limits_{t\to \infty }\,{\bf{e}}(t)=\mathop{\mathrm{lim}}\limits_{t\to \infty }\,\parallel {{\bf{y}}}_{1}(t)-{{\bf{y}}}_{2}(t)\parallel =0.$$

The drive-response model^51^ and the active-passive decomposition (APD) model^48–50^ are two widely used methods for characterizing the complete synchronization of chaotic systems. In the former model, the drive and response systems, which are to be synchronized, are in the unidirectional-coupling regime. It is required that the response system can be decomposed into a stable subsystem and an unstable one. By controlling the motion of the unstable subsystem, the driving part can force the phase-space orbit of the response part to reach a synchronized state. However, the drive-response model can only be applied to decomposable chaotic systems. This seriously restricts its applications in engineering. The APD model, as an advanced version of the drive-response model, provides a more general way to study complete synchronization. In the APD model, two chaotic parts to be synchronized can be written as the non-autonomous form:

3 $${\dot{{\bf{z}}}}_{1}={\bf{g}}[{{\bf{z}}}_{1},{\bf{s}}(t)],\,{\dot{{\bf{z}}}}_{2}={\bf{g}}[{{\bf{z}}}_{2},{\bf{s}}(t)],$$

where the temporal evolutions of **z**_1_ and **z**_2_ are ruled by the function **g**, and **s**(*t*) is the common external driving governed by the autonomous function $\dot{{\bf{s}}}(t)={\bf{h}}[{\bf{s}}(t)]$. The synchronization of these two chaotic systems depends on their error equation, which is given by

4 $$\dot{e}={\bf{g}}[{{\bf{z}}}_{1},{\bf{s}}(t)]-{\bf{g}}[{{\bf{z}}}_{1}-e,{\bf{s}}(t)],$$

where $e={{\bf{z}}}_{1}-{{\bf{z}}}_{2}$ is the synchronization error. Synchronization occurs if the error equation in Eq. (4) is asymptotically stable at the zero point $e=0$. The APD model provides a flexible method to find a proper function ${\bf{h}}[{\bf{s}}(t)]$ for the complete synchronization of chaotic systems. In this section, we use the APD model to study the chaotic synchronization of the two optical cavity modes in an optomechanical system.

**Figure 1:**
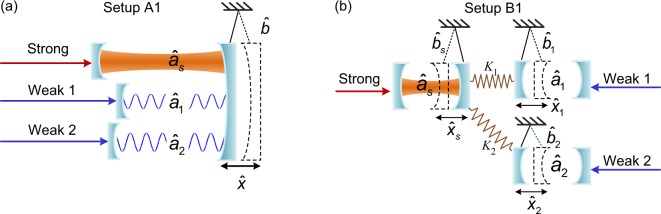
Schematic diagrams of two optomechanical models for complete synchronization. (**a**) Setup A1 includes a strongly driven cavity mode ${\hat{a}}_{s}$, two weakly driven cavity modes ${\hat{a}}_{j}$, and a mechanical mode $\hat{b}$. The strongly and weakly driven cavity modes are coupled via the mechanical mode with coupling strengths ${g}_{s}$ and ${g}_{j}$, respectively. (**b**) Setup B1 consists of a strongly driven cavity mode ${\hat{a}}_{s}$ and two weakly driven cavity modes ${\hat{a}}_{j}$, where the latter are coupled to ${\hat{a}}_{s}$ via the mechanical modes ${\hat{b}}_{j}$ and ${\hat{b}}_{s}$, which are additional coupled to each other with the spring coefficients ${k}_{j}$.

The setups are shown in Fig. 1, both of them consist of three subsystems: (i) a strongly driven cavity mode, ${\hat{a}}_{s}$; (ii) two weakly driven cavity modes, ${\hat{a}}_{1}$ and ${\hat{a}}_{2}$; (iii) mechanical mode(s), either *b* in setup A1, or ${\hat{b}}_{s}$, ${\hat{b}}_{1}$, and ${\hat{b}}_{2}$ in setup B1. The only difference is that the optical modes are integrated in one or different optomechanical resonators. In each setup, the cavity mode ${\hat{a}}_{s}$ is strongly driven by a single-frequency laser field to induce chaos. This chaos can be then transferred to the two weakly driven cavity modes (${\hat{a}}_{1}$ and ${\hat{a}}_{2}$) via the mechanical resonator(s)^23^. This chaos transferring regime is exactly the same as that in ref.^23^, which theoretically proves that the spectrum ${\tilde{S}}_{pump}(\omega )$ of the strongly driven optical cavity mode is proportional to that ${\tilde{S}}_{probe}(\omega )$ of the weakly driven optical mode: ${\tilde{S}}_{pump}(\omega )=\tilde{G}{\tilde{S}}_{probe}(\omega )$, where $\tilde{G}$ is a coefficient depending mainly on the coupling strength between the strongly (weakly) driven cavity and the mechanical resonator, and the corresponding field density. It is subsequently derived that the weakly driven cavities are chaotic if the strongly driven cavity is in the chaotic regime.

According to APD model, the mechanical oscillation corresponds to an external signal and the weakly driven cavity modes are the two subsystems to be synchronized. Analytically, we find that complete synchronization is stable to the mechanical input. We also numerically show in Sec. IV that the two weakly driven optical modes can be excited to chaotic states and can evolve into a completely synchronized state.

For simplicity, we neglect both thermal noise and quantum noise. This is valid under the following assumptions: (i) the thermal occupation of the cooled mechanical resonators is low, such that the thermal noise of the mechanical oscillators is small in comparison with the motion caused by the applied driving; (ii) the optomechanical system is driven by strong laser fields and, therefore, can be treated as a classical system. Under these conditions, the effect of environmental thermal noise and quantum noise of our optomechanical system can be neglected.

### Complete synchronization in setup A1

We start our discussion of complete synchronization by introducing setup A1, shown in Fig. 1(a). One strongly and two weakly driven cavity modes are coupled to the same mechanical mode. Here the strongly driven optical mode creates mechanical chaos through nonlinear optomechanical coupling. In this arrangement, the fields in the weakly driven cavity modes are modulated in a chaotic way by the chaotic mechanical mode. The total Hamiltonian of this synchronized system is given by (we set $\hslash =1$ and always assume $j=1,2$):

5 $$\begin{array}{rcl}\hat{H} & = & {\Delta }_{s}{\hat{a}}_{s}^{\dagger }{\hat{a}}_{s}+\sum _{j}\,{\Delta }_{j}{\hat{a}}_{j}^{\dagger }{\hat{a}}_{j}+{\Omega }_{m}{\hat{b}}^{\dagger }\hat{b}+i{\varepsilon }_{s}({\hat{a}}_{s}^{\dagger }-{\hat{a}}_{s})\\  &  & +\,i\,\sum _{j}\,{\varepsilon }_{j}({\hat{a}}_{j}^{\dagger }-{\hat{a}}_{j})+{g}_{s}{\hat{a}}_{s}^{\dagger }{\hat{a}}_{s}(\hat{b}+{\hat{b}}^{\dagger })+\sum _{j}\,{g}_{j}{\hat{a}}_{j}^{\dagger }{\hat{a}}_{j}(\hat{b}+{\hat{b}}^{\dagger }),\end{array}$$

where ${\hat{a}}_{s}$ (${\hat{a}}_{j}$) denotes the annihilation operator of the strongly (weakly) driven cavity mode, ${\Delta }_{s}={\omega }_{{\rm{cav}},s}-{\omega }_{d,s}$ (${\Delta }_{j}={\omega }_{{\rm{cav}},j}-{\omega }_{d,j}$) stands for the corresponding detuning between the cavity resonance frequency ${\omega }_{{\rm{cav}},s}$ (${\omega }_{{\rm{cav}},j}$) and the input laser frequency ${\omega }_{d,s}$ (${\omega }_{d,j}$), and ${\varepsilon }_{s}$ (${\varepsilon }_{j}$) is the driving strength of the cavity mode ${\hat{a}}_{s}$ (${\hat{a}}_{j}$). The annihilation operator of the mechanical resonator is represented by $\hat{b}$, and ${\Omega }_{m}$ denotes its natural frequency. Here, *g*_*s*_ (*g*_*j*_) is the optomechanical single-photon coupling strength between the cavity mode ${\hat{a}}_{s}$ (${\hat{a}}_{j}$) and the mechanical mode $\hat{b}$.

To obtain the equation of motion of the system in the classical regime, we first write the quantum Langevin equations for the Hamiltonian, given in Eq. (5), as:

6a $${\dot{\hat{a}}}_{s}=-\,i{\Delta }_{s}{\hat{a}}_{s}-\frac{{\gamma }_{s}}{2}{\hat{a}}_{s}-i{g}_{s}{\hat{a}}_{s}({\hat{b}}^{\dagger }+\hat{b})+{\varepsilon }_{s},$$

6b $$\dot{\hat{b}}=-\,i{\Omega }_{m}\hat{b}-\frac{{\Gamma }_{m}}{2}\hat{b}-i{g}_{s}{\hat{a}}_{s}^{\dagger }{\hat{a}}_{s}-i{g}_{j}{\hat{a}}_{j}^{\dagger }{\hat{a}}_{j},$$

6c $${\dot{\hat{a}}}_{j}=-\,i{\Delta }_{j}{\hat{a}}_{j}-\frac{{\gamma }_{j}}{2}{\hat{a}}_{j}-i{g}_{j}{\hat{a}}_{j}({\hat{b}}^{\dagger }+\hat{b})+{\varepsilon }_{j},$$

where *γ*_*s*_ (*γ*_*j*_) and ${\Gamma }_{m}$ are the damping rates of the cavity mode ${\hat{a}}_{s}$ (${\hat{a}}_{j}$) and the mechanical mode $\hat{b}$, respectively.

We treat the optomechanical device as a classical system such that we can replace the quantum operators with their classical mean values: ${\alpha }_{s}=\langle {\hat{a}}_{s}\rangle$, ${\alpha }_{j}=\langle {\hat{a}}_{j}\rangle$, and $\beta =\langle \hat{b}\rangle$. Note that the thermal noise and quantum noise are neglected, as explained above. Here, the back-action from the weakly driven cavity modes *α*_*j*_ on the mechanical mode *β* can be neglected, as it is comparably small to the strongly driven one. We denote this as the unidirectional-coupling regime, and in which the motion of the cavity mode *a*_*s*_ and the mechanical resonator are reduced to

7a $${\dot{\alpha }}_{s}=-\,i{\Delta }_{s}{\alpha }_{s}-\frac{{\gamma }_{s}}{2}{\alpha }_{s}-i{G}_{s}{\alpha }_{s}x+{\varepsilon }_{s},$$

7b $${m}_{{\rm{eff}}}\ddot{x}=-\,{m}_{{\rm{eff}}}{\Omega }_{m}^{2}x-{m}_{{\rm{eff}}}{\Gamma }_{m}\dot{x}+\hslash {G}_{s}|{\alpha }_{s}{|}^{2},$$

where *m*_eff_ denotes the effective mass of the mechanical resonator. An optomechanical system, governed by Eq. (7), can exhibit chaotic behavior^21–23,27–29^. Chaos originates from the nonlinear coupling between the cavity and mechanical modes, and can be transferred to the weakly driven cavity mode via the mechanical mode [Eq. (8)].

In this configuration, *α*_*j*_ are the two classical cavity modes to be synchronized, which are governed by

8 $${\dot{\alpha }}_{j}=-\,i{\Delta }_{j}{\alpha }_{j}-\frac{{\gamma }_{j}}{2}{\alpha }_{j}-i{G}_{j}{\alpha }_{j}x+{\varepsilon }_{j},$$

where $x={x}_{{\rm{ZPF}}}(\beta +{\beta }^{\ast })$ refers to the classical mechanical displacement, and its nonlinear coupling strength with the optical mode *α*_*j*_ is denoted by ${G}_{j}={g}_{j}/{x}_{{\rm{ZPF}}}$. Here *x*_ZPF_ is the zero-point fluctuation (ZPF) displacement of the mechanical resonator.

Let $e={\alpha }_{1}-{\alpha }_{2}$ be the synchronization error. Then from Eq. (8), we obtain the equation of motion for *e*,

9 $$\dot{e}=-\,i{\Delta }_{1}e-\frac{{\gamma }_{1}}{2}e-i{G}_{1}ex(t).$$

Note that the parameters of two weakly driven cavity modes are identical, i.e.: ${\Delta }_{1}={\Delta }_{2}$, ${\gamma }_{1}={\gamma }_{2}$, and ${G}_{1}={G}_{2}$. Complete synchronization occurs if the synchronization error system, described by Eq. (9), is asymptotically stable at the zero point. By constructing a strict Lyapunov function $V=\parallel {\bf{e}}{\parallel }^{2}\ge 0$, where ${\bf{e}}=[{\rm{Re}}(e),{\rm{Im}}(e)]$, we show that this system meets the criteria of uniformly asymptotical stability^52^, i.e., $\partial V/\partial t+(\partial V/\partial {\rm{e}})\,(\dot{{\bf{e}}})=-\,2\gamma \parallel {\bf{e}}{\parallel }^{2}\le 0$ and $V[{\bf{e}}(t+T),t+T]-V[{\bf{e}}(t),t]\le -\varUpsilon [\parallel {\bf{e}}(t)\parallel ]$ by letting $T=1/{\gamma }_{j}$ and $\varUpsilon [\parallel {\bf{e}}(t)\parallel ]=(1/2)\parallel {\bf{e}}(t){\parallel }^{2}$. This implies that the synchronization error decreases to zero as the time goes to infinity, and complete synchronization can be achieved in two chaotic identical optical modes.

### Complete synchronization in setup B1

In this subsection, we focus on setup B1 shown in Fig. 1(b). Specifically, this system consists of one strongly and two weakly driven optomechanical systems, each of which includes only a single cavity mode and a mechanical mode. Different from setup A1, here the optomechanical systems are coupled with each other via the mechanical resonators: each mechanical mode ${\hat{b}}_{j}$ in the weakly driven optomechanical system is coupled to the mechanical mode ${\hat{b}}_{s}$ in the strongly driven optomechanical system with a coupling coefficient *k*_*j*_. Moreover, in this setup, all the optomechanical systems are in the classical regime, such that the quantum operators of the cavity (${\hat{a}}_{s}$, ${\hat{a}}_{j}$) and mechanical modes (${\hat{b}}_{s}$, ${\hat{b}}_{j}$) can be replaced by their classical mean values: *α*_*s*_, *α*_*j*_, *β*_*s*_, and *β*_*j*_. Here, the equations of motion of the two weakly driven optomechanical systems are

10a $${\dot{\alpha }}_{j}=-\,i{\Delta }_{j}{\alpha }_{j}-\frac{{\gamma }_{j}}{2}{\alpha }_{j}+i{G}_{j}{\alpha }_{j}{x}_{j}+{\varepsilon }_{j},$$

10b $${m}_{{\rm{meff}},j}{\ddot{x}}_{j}=-\,{m}_{{\rm{meff}},j}{\Omega }_{j}^{2}{x}_{j}-{m}_{{\rm{meff}},j}{\Gamma }_{j}{\dot{x}}_{j}+\hslash {G}_{j}|{\alpha }_{j}{|}^{2}-{K}_{j}({x}_{j}-{x}_{s}).$$

These two system, described in Eq. (10), are driven by the same strongly driven optomechanical system:

11a $${\dot{\alpha }}_{s}=-\,i{\Delta }_{s}{\alpha }_{s}-\frac{{\gamma }_{s}}{2}{\alpha }_{s}-i{G}_{s}{\alpha }_{s}{x}_{s}+{\varepsilon }_{s},$$

11b $${m}_{{\rm{meff}},s}{\ddot{x}}_{s}=-\,{m}_{{\rm{meff}},s}{\Omega }_{s}^{2}{x}_{s}-{m}_{{\rm{meff}},s}{\Gamma }_{s}{\dot{x}}_{s}+\hslash {G}_{s}|{\alpha }_{s}{|}^{2},$$

where Δ_*s*_ (Δ_*j*_), *γ*_*s*_ (*γ*_*j*_), and ${\varepsilon }_{s}$ (${\varepsilon }_{j}$) refer to the detuning frequency, the damping rate, and the driving strength of the cavity mode in the strongly (weakly) driven optomechanical system. While ${m}_{{\rm{meff}},s}$ (${m}_{{\rm{meff}},j}$), ${\Omega }_{s}$ (${\Omega }_{j}$), ${\Gamma }_{s}$ (${\Gamma }_{j}$), and *x*_*s*_ (*x*_*j*_) are the mass, the resonance frequency, the damping rate, and the displacement of the mechanical mode ${\hat{b}}_{s}$ (${\hat{b}}_{j}$) of the strongly (weakly) driven optomechanical resonator, respectively. Also, its optomechanical coupling strength is denoted by *G*_*s*_ (*G*_*j*_). Here, *x*_*s*_ (*x*_*j*_) is defined as ${x}_{s}={x}_{{\rm{ZPF}}}^{s}({\beta }_{s}+{\beta }_{s}^{\ast })$ [${x}_{j}={x}_{{\rm{ZPF}}}^{j}({\beta }_{j}+{\beta }_{j}^{\ast })$], and we define ${G}_{s}={g}_{s}/{x}_{{\rm{ZPF}}}^{s}$ (${G}_{j}={g}_{j}/{x}_{{\rm{ZPF}}}^{j}$), where ${x}_{{\rm{ZPF}}}^{s}$ (${x}_{{\rm{ZPF}}}^{j}$) is the zero-point fluctuation of the strongly (weakly) driven optomechanical resonators and *g*_*s*_ (*g*_*j*_) refers to the single photon optical-mechanical coupling strength. The external force acting on the mechanical resonator associated with the displacement *x*_*j*_ takes the form $-{K}_{j}({x}_{j}-{x}_{s})$, where ${K}_{j}=\hslash {k}_{j}/({x}_{{\rm{ZPF}}}^{s}{x}_{{\rm{ZPF}}}^{j})$ is the classical mechanical coupling strength. When ${K}_{1}/{m}_{{\rm{eff}},1}={K}_{2}/{m}_{{\rm{meff}},2}$, the two weakly driven modes share the same dynamics. Thus, this system can be studied in the framework of the active-positive decomposition (APD) configuration: two weakly driven optomechanical resonators as two chaotic subsystems are synchronized and the strongly driven optomechanical resonator acts as a common external force.

In the mechanical strong-coupling regime, the mathematical model of setup B1 can be reduced to that of setup A1. In this case, the Lyapunov function in setup A1 is also valid, and complete synchronization is stable in this setup. Note that the external-force term $-{K}_{j}({x}_{j}-{x}_{s})$ in Eq. (11b) is derived from the classical Lagrangian ${L}_{{\rm{int}}}={K}_{j}{({x}_{j}-{x}_{s})}^{2}/2$. If we start from the quantum Langevin equations of the system, then the external-force term should be ${K}_{j}{x}_{s}$. This difference $-{K}_{j}{x}_{j}$ between these two functions originates from the quantization of classical coupled-spring oscillators. Quantum systems interact with each other in the discontinuous regime, while the classical ones interact in the continuous regime. When ${\Omega }_{j}^{2}\gg {K}_{j}$, the term $-{K}_{j}{x}_{j}$ in Eq. (10b) is very small when compared to other terms and can be omitted. Thus, Eq. (10b) and the corresponding quantum Langevin equation are consistent for high-frequency resonators.

To summarize, complete synchronization in setups A1 and B1 is a result of (i) dissipation of the cavities and (ii) a common external force. The chaotic mechanical resonator, working as a common force, adjusts the rhythms of the two optical modes to the same chaotic oscillation, while the dissipation of the cavities eliminate their initial state differences to reach a synchronized state.

In this section, we discussed the complete synchronization of two identical chaotic optical modes in optomechanical systems. However, it is technically challenging to fabricate two identical optomechanical resonators. Even a tiny parameter mismatch between two chaotic optomechanical resonators can destroy their complete synchronization. Thus, the research of synchronization in nonidentical chaotic systems is of importance. So far, there are many attempts for synchronization of two nonidentical chaotic systems, including phase synchronization^53,54^, generalized synchronization^55,56^, and time-delayed synchronization^57^. Below we describe phase synchronization of two nonidentical optical modes in an optomechanical system.

## Phase Synchronization

Now, we discuss in detail the synchronization of nonidentical chaotic cavity modes. In this section, we show that the phases of two nonidentical chaotic cavity modes can be locked at a fixed ratio, although their amplitudes are irrelevant to each other.

In general, two oscillators are called phase synchronized if their phases ${\psi }_{1}(t)$ and ${\psi }_{2}(t)$ are locked at a fixed ratio *m*/*n*, i.e., $|n{\psi }_{1}(t)-m{\psi }_{2}(t)| < {\rm{constant}}$, where *m* and *n* are integers. This notion of phase synchronization has been extended to chaotic systems. We find that two weakly-coupled chaotic systems can be perfectly phase locked even if their amplitudes are irrelevant. Note that the phase in this phase synchronization is essentially different from the phases of complex fields in setups A1 and B1, and is used here to describe the rotation of the orbit of a chaotic system. The definition of this phase in a chaotic system is not unique. Indeed, various versions have been proposed based on analytic signal processing methods^58^ or the Poincaré section. Here, we use the former to study the phase synchronization of chaotic cavities. To obtain the temporal phase, observed in an arbitrary-scale time function *s*(*t*), a complex analytic signal $\phi (t)$ is reconstructed from *s*(*t*), i.e.,

12 $$\phi (t)=s(t)+i\tilde{s}(t)=A(t)\,\exp [i\Psi (t)],$$

where *A*(*t*) is the amplitude of the signal and $\Psi (t)$ is its phase, while $\tilde{s}(t)$ is the Hilbert transform of *s*(*t*), which is given by

13 $$\tilde{s}(t)=\frac{1}{\pi }{\rm{P}}.{\rm{V}}.\,{\int }_{-\infty }^{\infty }\,\frac{s(\tau )}{t-\tau }d\tau ,$$

where P.V. denotes the Cauchy principal value. Note that the phases are unwrapped, i.e., these are not constrained to the range $(\,-\,\pi ,\pi ]$. Once *s*(*t*) is obtained, the amplitude *A*(*t*) and phase $\Psi (t)$ can be calculated^58^.

In this section, we also study the configuration mentioned in Secs 1 and 2: The mechanical resonator acts on the frequency term of each chaotic cavity mode, and modulates their rotations in the same way. More specifically, in the small-detuning strong-coupling regime, the mechanical resonator governs the rotation of each chaotic cavity mode with different coupling strengths. These cavity modes can, thus, be locked at a fixed value associated with the coupling strengths. Here, the optical rotations are measured by the wrapped phases calculated by Eq. (13).

**Figure 2:**
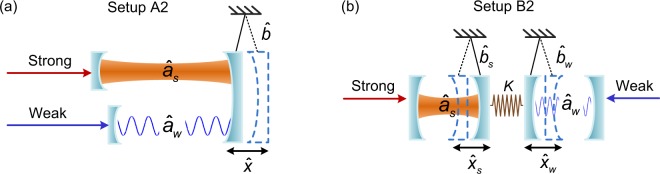
Schematic diagrams of two different setups for phase synchronization. (**a**) Setup A2 consists of a strongly driven optical mode ${\hat{a}}_{s}$, a weakly driven optical mode ${\hat{a}}_{w}$, and a mechanical mode $\hat{b}$. Both the optical modes are coupled to the mechanical resonator and integrated into a single optomechanical system; (**b**) Setup B2 includes one strongly driven (${\hat{a}}_{s}$ and ${\hat{b}}_{s}$) and one weakly driven (${\hat{a}}_{w}$ and ${\hat{b}}_{w}$) optomechanical systems, which are coupled via the mechanical modes ${\hat{b}}_{s}$ and ${\hat{b}}_{w}$ with a coupling coefficient *K*.

For examples, we consider the phase synchronization of strongly and weakly driven cavity modes. The setups are shown in Fig. 2(a,b), both of them consist of one strongly driven mode, one weakly driven cavity mode, and one or more mechanical mode(s). we describe a configuration for phase synchronization of a strongly driven cavity mode and a weakly driven one in an optomechanical system. When the driving-enhanced optomechanical coupling is strong and the cavity-driving detuning is small, i.e., in the strong optical-mechanical coupling and small-detuning regime, the temporal phase of each optical mode mainly depends on the displacement(s) of the mechanical resonator(s). In this configuration, the two chaotic optical cavity modes can be prepared in phase synchronized, regardless of their amplitudes. As in complete synchronization, here we also neglect thermal noise and quantum noise.

The detailed description of these two setups is presented in the following subsections.

### Phase synchronization in setup A2

As shown in Fig. 2(a), the system consists of a strongly driven cavity mode ${\hat{a}}_{s}$ and a weakly driven cavity mode ${\hat{a}}_{w}$, and a mechanical mode $\hat{b}$ associated with displacement $\hat{x}={x}_{{\rm{ZPF}}}({\hat{b}}^{\dagger }+\hat{b})$. These two cavity modes ${\hat{a}}_{s}$ and ${\hat{a}}_{w}$ are coupled to the mechanical mode $\hat{b}$ in the unidirectional-coupling regime. Next we show how the phases of the two chaotic optical modes, in the strongly and weakly driven regimes in setup A2, can be locked at a fixed ratio.

Let *α*_*s*_, *α*_*w*_, and *β* be the mean values of ${\hat{a}}_{s}$, ${\hat{a}}_{w}$, and $\hat{b}$: ${\alpha }_{s}=\langle {\hat{a}}_{s}\rangle$, ${\alpha }_{w}=\langle {\hat{a}}_{w}\rangle$, and $\beta =\langle \hat{b}\rangle$ in the classical regime. Their dynamics is governed by:

14a $${\dot{\alpha }}_{s}=-\,i{\Delta }_{s}{\alpha }_{s}-\frac{{\gamma }_{s}}{2}{\alpha }_{s}-i{G}_{s}{\alpha }_{s}x+{\varepsilon }_{s},$$

14b $${\dot{\alpha }}_{w}=-\,i{\Delta }_{w}{\alpha }_{w}-\frac{{\gamma }_{w}}{2}{\alpha }_{w}-i{G}_{w}{\alpha }_{w}x+{\varepsilon }_{w},$$

where ${\Delta }_{s}={\omega }_{{\rm{cav}},s}-{\omega }_{d,s}$ (${\Delta }_{w}={\omega }_{{\rm{c}}av,w}-{\omega }_{d,w}$) is the corresponding detuning between the cavity resonance frequency ${\omega }_{{\rm{cav}},s}$ (${\omega }_{{\rm{cav}},w}$) and the laser frequency ${\omega }_{d,s}$ (${\omega }_{d,w}$), while ${\varepsilon }_{s}$ (${\varepsilon }_{w}$) and *γ*_*s*_ (*γ*_*w*_) are the driving strength and the damping rates of the strongly (weakly) driven cavity mode ${\hat{a}}_{s}$ (${\hat{a}}_{w}$), respectively. Here, ${G}_{s}={g}_{s}/{x}_{{\rm{ZPF}}}^{s}$ (${G}_{w}={g}_{w}/{x}_{{\rm{ZPF}}}^{w}$) represents the coupling strength between the strongly (weakly) driven cavity mode ${\hat{a}}_{s}$ (${\hat{a}}_{w}$) and the mechanical mode *β*, where ${x}_{{\rm{ZPF}}}^{s}$ (${x}_{{\rm{ZPF}}}^{w}$) is the zero-point fluctuation of the strongly (weakly) driven optomechanical resonators and *g*_*s*_ (*g*_*w*_) refers to the optomechanical single-photon coupling strength between the cavity mode ${\hat{a}}_{s}$ (${\hat{a}}_{w}$) and the mechanical mode $\hat{b}$.

The cavity modes *α*_*s*_ and *α*_*w*_ are two parts to be synchronized. They are driven by the same mechanical mode *β*. The mechanical motion is governed by

15 $${m}_{{\rm{eff}}}\ddot{x}=-\,{m}_{{\rm{eff}}}{\Omega }_{m}^{2}x-{m}_{{\rm{eff}}}{\Gamma }_{m}\dot{x}+\hslash {G}_{s}|{\alpha }_{s}{|}^{2},$$

where ${\Omega }_{m}$ is the resonance frequency of the mechanical resonator mode $\hat{b}$, and ${\Gamma }_{m}$ is its damping rate. In our arrangement, the effects of the weakly driven optical modes acting on the mechanical mode *x* can be neglected by choosing ${G}_{s}|{\alpha }_{s}{|}^{2}\gg {G}_{w}|{\alpha }_{w}{|}^{2}$.

Next, we find the relation of parameters determining the ratio of the unwrapped phase of cavity modes in phase synchronization. We define the mean value of the mechanical displacement *x* as $\bar{x}={\mathrm{lim}}_{t\to \infty }\,{(t-{t}_{0})}^{-1}\,{\int }_{{t}_{0}}^{t}\,|x(t^{\prime} )|dt^{\prime}$, where *t*_0_ is the initial time. We refer to the conditions ${G}_{s}\bar{x}\gg {\Delta }_{s}$ (${G}_{w}\,\bar{x}\gg {\Delta }_{w}$) and ${G}_{s}\bar{x}\gg {\gamma }_{s}$ (${G}_{w}\,\bar{x}\gg {\gamma }_{w}$) as the strong-coupling small-detuning regime. In this regime, the instantaneous frequencies of both strongly and weakly driven optical modes are determined by the following two factors: the detuning Δ_*s*_ (Δ_*w*_) and the mechanical displacement-dependent parameter ${G}_{s}x$ (${G}_{w}x$). For on-resonance driving, ${\Delta }_{s}={\Delta }_{w}\approx 0$, the evolution of the strongly (weakly) driven optical mode *α*_*s*_ (*α*_*w*_) depends mainly on the mechanical motion ${G}_{s}\bar{x}$ (${G}_{w}\bar{x}$). Thus, the unwrapped phases, ${\Psi }_{w}(t)$ and ${\Psi }_{s}(t)$ of the cavity modes *α*_*s*_ and *α*_*w*_, defined in Eq. (12) are locked at a fixed ratio of

16 $$\mathop{\mathrm{lim}}\limits_{{\rm{t}}\to \infty }\frac{{\Psi }_{w}(t)}{{\Psi }_{s}(t)}=\frac{{G}_{w}}{{G}_{s}},$$

as the time approaches infinity.

To conclude, in the strong-coupling small-detuning regime, the mechanical displacement *x* dominates the unwrapped phases of the two chaotic optical modes (*α*_*s*_ and *α*_*w*_). While the coupling strengths (*G*_*s*_ and *G*_*w*_) act as the weighting factors of *x* in this process. Therefore, the unwrapped phases of the two chaotic optical modes (*α*_*s*_ and *α*_*w*_) can be locked at a fixed value related to *G*_*s*_ and *G*_*w*_.

### Phase synchronization in setup B2

As shown in Fig. 2(b), the setup B2 consists of a strongly driven optomechanical system and a weakly driven one, each of which includes only a single cavity mode and a mechanical mode. Different from setup A2, two optomechanical resonators are mechanically coupled with each other with a coupling coefficient *k*. Treating the whole system classically, we can replace the operators ${\hat{a}}_{s}$ (${\hat{a}}_{w}$) of the quantum cavity modes and ${\hat{b}}_{s}$ (${\hat{b}}_{w}$) of the quantum mechanical resonator mode for the strongly (weakly) driven optomechanical system with their mean values: ${\alpha }_{s}=\langle {\hat{a}}_{s}\rangle$, ${\alpha }_{w}=\langle {\hat{a}}_{w}\rangle$, ${\beta }_{s}=\langle {\hat{b}}_{s}\rangle$, and ${\beta }_{w}=\langle {\hat{b}}_{w}\rangle$.

The dynamics of the weakly driven optomechanical resonator is described by:

17a $${\dot{\alpha }}_{w}=-\,i{\Delta }_{w}{\alpha }_{w}-\frac{{\gamma }_{w}}{2}{\alpha }_{w}+i{G}_{w}{\alpha }_{w}{x}_{w}+{\varepsilon }_{w},$$

17b $${m}_{w}{\ddot{x}}_{w}=-\,{m}_{w}{\Omega }_{w}^{2}{x}_{w}-{m}_{w}{\Gamma }_{w}{\dot{x}}_{w}+\hslash {G}_{w}|{\alpha }_{w}{|}^{2}-K({x}_{w}-{x}_{s}).$$

In the unidirectional coupling regime, the motion of the weakly driven optomechanical part is governed by the strongly driven optomechanical one. The motion of the latter can be modeled as

18a $${\dot{\alpha }}_{s}=-\,i{\Delta }_{s}{\alpha }_{s}-\frac{{\gamma }_{s}}{2}{\alpha }_{s}-i{G}_{s}{\alpha }_{s}{x}_{s}+{\varepsilon }_{s},$$

18b $${m}_{s}{\ddot{x}}_{s}=-\,{m}_{s}{\Omega }_{s}^{2}{x}_{s}-{m}_{s}{\Gamma }_{s}{\dot{x}}_{s}+\hslash {G}_{j}|{\alpha }_{s}{|}^{2},$$

where Δ_*s*_ (Δ_*w*_), *γ*_*s*_ (*γ*_*w*_), and ${\varepsilon }_{s}$ (${\varepsilon }_{w}$) are the corresponding detuning, damping rate, and driving strength of the cavity mode in the strongly (weakly) driven optomechanical resonator. While the effective mass, resonance frequency, and mechanical damping rate of the mechanical mode ${\hat{b}}_{s}$ (${\hat{b}}_{w}$) are represented by ${m}_{{\rm{eff}},s}$ (${m}_{{\rm{eff}},w}$), ${\Omega }_{s}$ (${\Omega }_{w}$) and ${\Gamma }_{s}$ (${\Gamma }_{w}$), respectively. Here, ${G}_{s}={g}_{s}/{x}_{{\rm{ZPF}}}^{s}$ (${G}_{w}={g}_{w}/{x}_{{\rm{ZPF}}}^{w}$) is the optical-mechanical coupling strength in the strongly (weakly) driven optomechanical part. The displacements of the strongly and weakly driven mechanical oscillators are given by ${x}_{s}={x}_{{\rm{ZPF}}}^{s}({\beta }_{s}+{\beta }_{s}^{\ast })$ and ${x}_{w}={x}_{{\rm{ZPF}}}^{w}({\beta }_{w}+{\beta }_{w}^{\ast })$, where ${x}_{{\rm{ZPF}}}^{s}$ and ${x}_{{\rm{ZPF}}}^{w}$ are the corresponding ZPF displacements of the left and right mechanical resonators.

In this setup, the term $-K({x}_{w}-{x}_{s})$ in Eq. (17) with a mechanical coupling strength $K=\hslash k/({x}_{{\rm{ZPF}}}^{s}{x}_{{\rm{ZPF}}}^{w})$ is the external force driving the mechanical mode *x*_*w*_. It provides a positive feedback to the weakly driven mechanical mode *x*_*w*_ when $({x}_{w}-{x}_{s}) < 0$. This feedback turns to be negative when $({x}_{w}-{x}_{s}) > 0$. Thus, when the coupling coefficient *k* is strong enough, we have the relation: ${x}_{w}(t)\approx {x}_{s}(t)$. We define the mean value of the mechanical displacement *x*_*m*_ as ${\bar{x}}_{m}={\mathrm{lim}}_{t\to \infty }{(t-{t}_{0})}^{-1}\,{\int }_{{t}_{0}}^{t}\,|{x}_{m}(t^{\prime} )|dt^{\prime}$, where *t*_0_ is the initial time and $m=s$ (*w*) stands for the strongly (weakly) driven mode. In the strong-coupling small-detuning regime when ${G}_{s}{\bar{x}}_{s}\gg {\Delta }_{s}$ (${G}_{w}{\bar{x}}_{w}\gg {\Delta }_{w}$) and ${G}_{s}{\bar{x}}_{s}\gg {\gamma }_{s}$ (${G}_{w}{\bar{x}}_{w}\gg {\gamma }_{w}$), the temporal phase of the strongly (weakly) driven optical mode *α*_*s*_ (*α*_*w*_) mainly depends on ${G}_{s}{\bar{x}}_{s}$ (${G}_{w}{\bar{x}}_{s}$). Under these approximations, the ratio of the unwrapped phases ${\Psi }_{w}(t)$ and ${\Psi }_{s}(t)$ of *α*_*s*_ and *α*_*w*_ for this setup B2 in the infinite-time limit is the same as the corresponding limit, given in Eq. (16), for setup A2.

We now discuss our idea for the chaotic synchronization of optomechanical systems in the unidirectional coupling regime. This treatment is reasonable as long as ${G}_{s}|{\alpha }_{s}{|}^{2}\gg {G}_{w}|{\alpha }_{w}{|}^{2}$. Note that both complete and phase synchronization can be obtained with a slight change in the chaotic motion of the mechanical resonators, when taking into account the weak force from the weakly driven cavity modes on the mechanical resonators.

## Results

In Sec. II, we presented four setups for both complete and phase synchronization of chaotic optical modes in an optomechanical system. These setups are different in their configurations but share a common dynamics: the strongly driven cavity mode overwhelms the weakly driven cavity modes and drives them into chaotic motion. For each setup, we plotted the corresponding phase portraits to show their dynamical motions. We assume that the strongly driven optomechanical resonators in each setup are in the red detuning regime, and their values are based on the experimental data given in ref.^29^. The values of the other parameters, which were set in our simulations, are also experimentally accessible with current technologies. Complete synchronization can be achieved when the amplitude of the strongly driven cavity field is much larger than the weakly driven ones: $|{\alpha }_{s}|\gg |{\alpha }_{1}|$ and $|{\alpha }_{s}|\gg |{\alpha }_{2}|$. While phase synchronization requires more conditions: ${G}_{s}\bar{x}\gg {\Delta }_{s}$ (${G}_{w}\bar{x}\gg {\Delta }_{w}$) and ${G}_{s}\bar{x}\gg {\gamma }_{s}$ (${G}_{w}\bar{x}\gg {\gamma }_{w}$). Now we present our numerical results below for the configurations of these two types of synchronization.

### Complete synchronization

As mentioned above, we propose two setups for realizing complete synchronization. Both setups are described by the APD model. The mechanical mode plays two key roles: (i) transferring chaos from the strongly driven cavity mode to the weakly driven ones, and (ii) acting as a common external force on the optical modes. Below we provide several numerical simulations for the complete synchronization of these two setups.

#### Complete synchronization in setup A1

**Figure 3:**
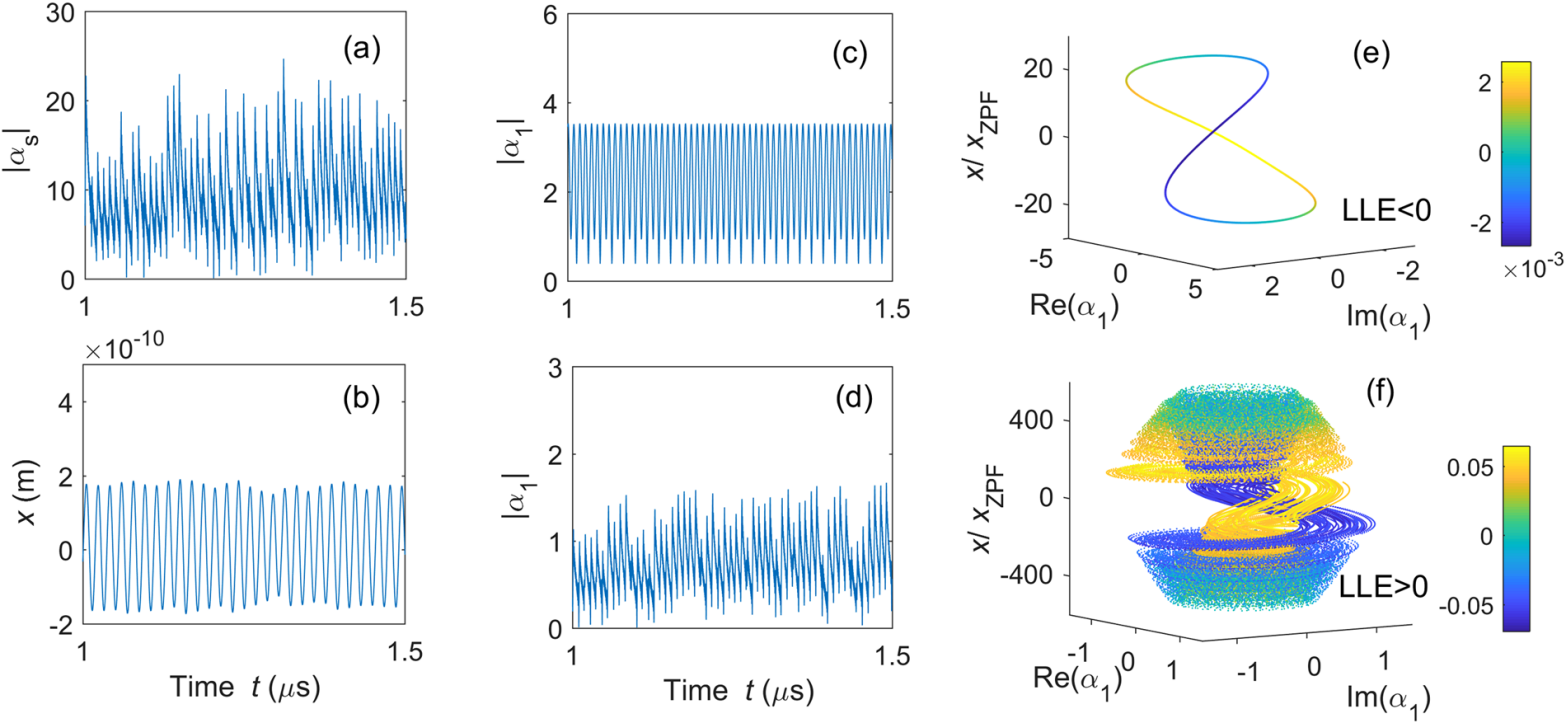
Complete synchronization in setup A1: Time evolutions for (**a**) $|{\alpha }_{s}|$, (**b**) $x$, and $|{\alpha }_{1}|$, (**c**) without and (**d**) with coupling to $|{\alpha }_{s}|$. While, (**e** and **f**) are the phase portraits of optical cavity mode ${\alpha }_{1}$ and the mechanical mode $\beta$ without and with coupling to ${\alpha }_{s}$. The parameters are set as: ${\Delta }_{1}/2\pi =13\,{\rm{MHz}}$, ${\gamma }_{1}/2\pi ={\gamma }_{s}/2\pi =0.24\,{\rm{GHz}}$, ${g}_{s}/2\pi =0.126\,{\rm{GHz}}$, ${\varepsilon }_{1}/2\pi =22\,{\rm{MHz}}$, ${\Delta }_{s}/2\pi =0.13\,{\rm{GHz}}$, ${\varepsilon }_{s}/2\pi =15.4\,{\rm{GHz}}$, ${\Gamma }_{m}/2\pi =2.8\,{\rm{MHz}}$, ${\Omega }_{m}/2\pi =0.346\,{\rm{GHz}}$. Moreover, ${g}_{1}/2\pi =0$ for (**c** and **e**), and ${g}_{1}/2\pi =0.126\,{\rm{GHz}}$ for (**d** and **f**). The largest Lyapunov exponent (LLE) is calculated to be (**e**) ${\rm{LLE}}/{\Omega }_{m}=-\,0.0002$ and (**f**) ${\rm{LLE}}/{\Omega }_{m}=1.97$. Here, ${\rm{Re}}({\alpha }_{1})$, ${\rm{Im}}({\alpha }_{1})$, and *x* correspond to the three coordinates of the three-dimensional phase space, and the fourth variable $\dot{x}$ in (**e** and **f**) is characterized in the color scale according to the depicted colorbar.

In setup A1, the system consists of three cavity modes (i.e., one strongly driven and two weakly driven modes) and a mechanical mode. Each cavity mode is coupled to each other via the mechanical mode. To realize chaotic synchronization, first we need to prepare the weakly driven cavity modes *α*_1_ and *α*_2_ in chaotic states. However, in general, weakly driven optomechanical systems can only generate nonchaotic fields. An efficient method to obtain a weak chaotic field is that connecting a weakly driven cavity mode to a chaotic resonator. In this setup, chaos is generated by the strongly driven cavity mode, and then transferred to the weakly driven cavity modes *α*_1_ and *α*_2_ via the mechanical mode^23^. Here, the cavity mode *α*_1_ is taken as an example to show how its dynamics transfers from regular into chaotic. To give a straightforward view of this transfer, we numerically calculate its phase portraits without [see Fig. 3(e)] and with [see Fig. 3(f)] the driving from the strongly driven optical mode. Moreover, we calculate the largest Lyapunov exponent (LLE) of the system with the method proposed in^59,60^ to check if *α*_1_ evolved to a chaotic state. A positive LLE is an indicator of chaos, while a non-positive LLE means regular motion.

To make a clear comparison of the weakly driven optical mode with and without coupling to the strongly driven optical mode, we first consider the case without the cavity mode ${\alpha }_{s}$. In this case, the optomechanical system is reduced to a single weakly driven optical mode *α*_1_ and a single mechanical mode ${\beta }_{1}$. As shown in Fig. 3(c), the time series of $|{\alpha }_{s}(t)|$ shows a regular fluctuation as the time progresses. Also, in the corresponding phase portrait [see Fig. 3(e)], a single closed loop is found with ${\rm{LLE}} < 0$, implying that the system is in regular periodic motion in the weakly driven regime. Then, we study the system shown in Fig. 1(a), in which the two weakly driven cavity modes are coupled to the strongly driven cavity mode via the mechanical mode. The time series of the amplitudes of the weakly driven cavity mode *α*_1_ is converted from regular [see Fig. 3(c)] to chaotic motion [see Fig. 3(d)] with the coupling of the strongly driven cavity resonator ${\alpha }_{s}$ [see Fig. 3(a)]. We find that the amplitude of *α*_1_ in Fig. 3(c) is even larger than the one in Fig. 3(d). This is because the generated photons are distributed in a broad range of frequencies when the cavity mode is in the chaotic regime, while they are concentrated in a single frequency for periodic cases. It can be seen from the phase portrait that a chaotic attractor appears even if it is weakly driven [see Fig. 3(f)]. We find that ${\rm{LLE}} > 0$. This means that the weakly driven optical mode is successfully driven to a chaotic state. In the chaotic regime, both optical and mechanical resonators are excited to very high values, such that the back-action effect during the measurements can be neglected in experiments. The phase portraits in Fig. 3(f) consist of two complex variables: the weakly driven cavity mode *α*_1_ and the mechanical mode $\beta$. For simplicity, we expand this two-dimensional complex space $({\alpha }_{1},\beta )$ to the four-dimensional real space [Re(*α*_1_), Im(*α*_1_), $x$, $\dot{x}$], where $x$ and $\dot{x}$ denote the displacement and the velocity of the mechanical mode, respectively. The value of $\dot{x}$ is presented as different colors.

**Figure 4:**
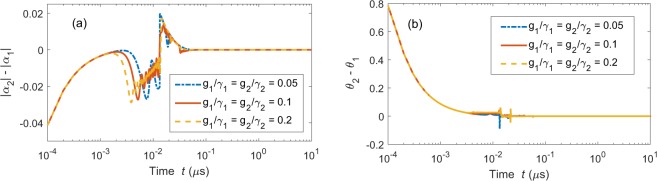
Synchronization errors for complete synchronization in setup A1: (**a**) amplitude errors and (**b**) phase errors between the two chaotic weakly driven cavity modes ${\alpha }_{1}$ and ${\alpha }_{2}$ as a function of time *t*. Here, ${g}_{1}/2\pi ={g}_{2}/2\pi =0.126\,{\rm{GHz}}$, ${\Delta }_{2}/2\pi =13\,{\rm{MHz}}$, ${\gamma }_{2}/2\pi =0.24\,{\rm{GHz}}$, and ${\varepsilon }_{2}/2\pi =22\,{\rm{MHz}}$. The initial conditions are set as: ${\alpha }_{1}(0)=0.1+0.1i$, ${\alpha }_{2}(0)=0.1i$, ${\alpha }_{s}(0)=0$, and $\beta (0)=0$. All the other parameters are the same as in Fig. 3.

We use the synchronization error between two chaotic fields ${\alpha }_{1}$ and ${\alpha }_{2}$ as the criterion of complete synchronization. The synchronization error includes the amplitude error $|{\alpha }_{2}|-|{\alpha }_{1}|$ and phase error ${\theta }_{2}(t)-{\theta }_{1}(t)$, where $|{\alpha }_{1}|$ ($|{\alpha }_{2}|$) is the amplitude of the cavity mode *α*_1_ (*α*_2_), and its phase is denoted by ${\theta }_{1}(t)$ [${\theta }_{2}(t)$]. The chaotic cavity fields *α*_1_ and *α*_2_ are completely synchronized if both of their amplitude and phase errors converge to zero as the evolution time progresses to infinity. Figure 4 shows the synchronization error between the two chaotic fields *α*_1_ and *α*_2_ for three different values of the coupling strengths ${g}_{1}$ and ${g}_{2}$. Note that the initial conditions of *α*_1_ and *α*_2_ are set to be different. In general, two neighboring chaotic trajectories without coupling will rapidly depart from each other because chaos is sensitive to initial conditions. However, we can find that both amplitude error $|{\alpha }_{2}(t)|-|{\alpha }_{1}(t)|$ [see Fig. 4(a)] and phase error ${\theta }_{2}(t)-{\theta }_{1}(t)$ [see Fig. 4(b)] decrease to zero after conquering the transient states. The jumps seen in Fig. 4(b) occur because of the sudden transitions of the chaotic orbits. Thus, complete synchronization is obtained in the two weakly driven cavity modes and this synchronization is independent of the coupling strengths ${g}_{1}$ and ${g}_{2}$. Note that if the coupling strength $g/{\gamma }_{1} < 0.05$, then the weakly driven cavity mode *α*_1_ cannot be driven to a chaotic state. In Figs 3(b) and 4(b), we show that the weakly driven cavity modes: (i) can be driven to the chaotic modes and (ii) can realize the synchronization with each other under the driving of the chaotic mechanical resonator.

#### Complete synchronization in setup B1

Now we study complete synchronization in setup B1, shown in Fig. 1(b). The system includes three optomechanical subsystems: two weakly driven optomechanical objects are coupled to the strongly driven optomechanical one via the mechanical coupling. In this subsection, we numerically show, by preparing the strongly driven optomechanical part in a chaotic state, that the weakly driven parts can also be driven into synchronized chaotic states.

**Figure 5:**
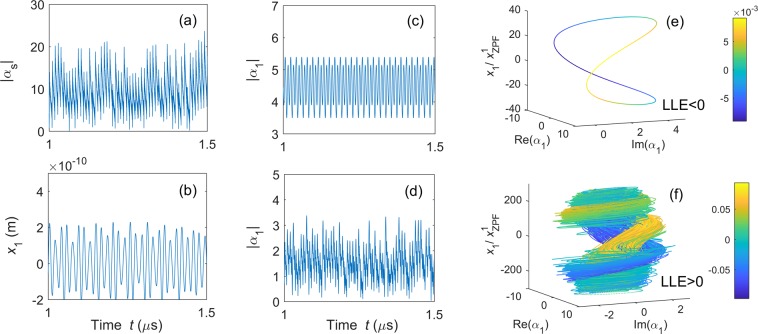
Complete synchronization in setup B1: Time evolutions of (**a**) $|{\alpha }_{s}|$, (**b**) ${x}_{1}$, and $|{\alpha }_{1}|$ (**c**) without and (**d**) with coupling to ${\alpha }_{s}$. While (**e** and **f**) correspond to the phase portraits of the weakly driven optomechanical system 1 (**e**) without and (**f**) with coupling to the strongly driven one. The parameters are: ${\Delta }_{1}/2\pi =26\,{\rm{MHz}}$, ${g}_{1}/2\pi =25.2\,{\rm{MHz}}$, ${\Gamma }_{s}/2\pi ={\Gamma }_{1}/2\pi =2.8\,{\rm{MHz}}$, ${\Omega }_{s}/2\pi ={\Omega }_{1}/2\pi =0.346\,{\rm{GHz}}$. Moreover, ${k}_{1}/2\pi =0\,{\rm{MHz}}$ for (**c** and **e**); and ${k}_{1}/2\pi =129\,{\rm{MHz}}$ for (**d** and **f**), while other parameters are the same as in Fig. 3. The largest Lyapunov exponent (LLE) is calculated as: (**a**) ${\rm{LLE}}/{\Omega }_{s}=-\,0.00007$ and (**b**) ${\rm{LLE}}/{\Omega }_{s}=1.97$. Here, the different colors in (**e** and **f**) correspond to the values of ${\dot{x}}_{1}$ shown in the colorbar.

Since the two weakly driven components share the same dynamics, we choose one of these as an example, and numerically calculate its temporal amplitudes and phase portraits with [Fig. 5(c,e)] and without [Fig. 5(d,f)] coupling to the strongly driven component. The results turn out to be similar to the ones in setup A1. Without the strongly driven part, the weakly driven component is in regular motion: the amplitude of the cavity mode, $|{\alpha }_{1}(t)|$, fluctuates periodically and the corresponding phase portrait shows a single loop. The periodic time series of $|{\alpha }_{1}(t)|$ becomes chaotic and the phase portrait turns to a chaotic attractor after the strongly driven optomechanical resonator is coupled. Moreover, this transition is also indicated by LLE, changing from negative in Fig. 5(e) to positive in Fig. 5(f). Similarly to Fig. 3, the complex two-dimensional weakly driven optomechanical resonator [${\alpha }_{1}$, ${\beta }_{1}$] is illustrated in the four-dimensional real space [Re(*α*_1_), Im(*α*_1_), ${x}_{1}$, ${\dot{x}}_{1}$]. Here, Re(*α*_1_) [Im(*α*_1_)] is the real (imaginary) part of the classical cavity mode *α*_1_, and ${x}_{1}$ (${\dot{x}}_{1}$) denotes the displacement (velocity) of the classical mechanical mode ${\beta }_{1}$. The color of the lines show the values of the fourth component ${\dot{x}}_{1}$.

**Figure 6:**
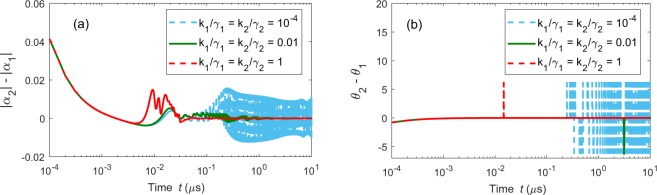
Synchronization errors for complete synchronization in setup B1: (**a**) amplitude errors and (**b**) phase errors between the cavity modes *α*_1_ and *α*_2_ as a function of time *t* for different mechanical-mechanical coupling coefficients ${k}_{1}$ and ${k}_{2}$ as a function of time *t*. The initial conditions of the weakly and strongly driven optomechanical systems are set as: $[{\alpha }_{1}(0),{\beta }_{1}(0)]=(0.1i,0)$, $[{\alpha }_{2}(0),{\beta }_{2}(0)]=(0.1+0.1i,0)$, and $[{\alpha }_{s}(0),{\beta }_{s}(0)]=(0,0)$. The parameters are: ${k}_{1}/2\pi ={k}_{2}/2\pi =129\,{\rm{MHz}}$, while all the other parameters are the same as in Fig. 5.

As discussed in setup A1, the two chaotic cavity modes ${\alpha }_{1}(t)$ and ${\alpha }_{2}(t)$ can achieve complete synchronization, if their error converges to zero. Figure 6(a,b) show the amplitude error, $|{\alpha }_{2}|-|{\alpha }_{1}|$, and phase error, ${\theta }_{2}(t)-{\theta }_{1}(t)$, for different mechanical-coupling coefficients ${k}_{1}$ and ${k}_{2}$, where $|{\alpha }_{1}|$ ($|{\alpha }_{2}|$) and ${\theta }_{1}(t)$ [${\theta }_{2}(t)$] denote the amplitude and phase of the cavity mode *α*_1_ (*α*_1_), respectively. The initial condition difference is set to be: $|{\alpha }_{2}(0)|-|{\alpha }_{1}(0)|=0.041$ in Fig. 6(a) and ${\theta }_{2}(t)-{\theta }_{1}(t)=-\,\pi /4$ in Fig. 6(b). When ${k}_{1}$ and ${k}_{2}$ are very weak (${k}_{1}/{\gamma }_{1}={k}_{2}/{\gamma }_{2}={10}^{-4}$), both amplitude and phase errors fluctuate considerably [blue dashed curves in Fig. 6(a,b)] as the evolution time progresses. When ${k}_{1}$ (${k}_{2}$) increases to ${k}_{1}/{\gamma }_{1}={k}_{2}/{\gamma }_{2}={10}^{-2}$ [green dashed dot curves in Fig. 6(a,b)], these two errors drastically fluctuate in the beginning, and then decrease to zero after a transient period. Moreover, the increase of the coupling strength ${k}_{1}$ (${k}_{2}$) accelerates the convergence of the synchronization errors, as shown in Fig. 6(a,b) (red solid curve). The time to reach synchronization greatly decreases as the parameters ${k}_{1}$ and ${k}_{2}$ increase to ${k}_{1}/{\gamma }_{1}={k}_{2}/{\gamma }_{2}=1$ from ${k}_{1}/{\gamma }_{1}={k}_{2}/{\gamma }_{2}={10}^{-2}$. Obviously, the mechanical-coupling parameters ${k}_{1}$ and ${k}_{2}$ play a crucial role in the synchronization of chaotic optical fields. Two weakly driven optomechanical systems can be driven into complete synchronization when the mechanical coupling ${k}_{1}$ and ${k}_{2}$ are large enough.

#### Comparison of setups A1 and B1

Complete synchronization can be realized in both setups A1 and B1 according to the APD model. As shown in Figs 4 and 6, the motions of the two weakly driven cavity modes tend to be close to each other and become completely identical as the time progresses. As our theoretical prediction, the weakly driven cavity modes in chaotic motion can be in complete synchronization if they are asymptotically stable and their motion is dominated by a common external force, which is the strongly driven cavity mode here. Chaos can be transferred from the strongly driven cavity mode to the weakly driven cavity modes by mediation of a direct coupling in setup A1 (see Fig. 3) or indirect coupling in setup B1 (see Fig. 5). In setup A1, the two weakly driven cavity modes are synchronized. They are driven by the same mechanical mode.

For setup A1, complete synchronization occurs due to the configuration that the two identical weakly-driven cavity modes are driven by the same mechanical mode. In this regime, the damping of the cavity modes removes the information about the initial cavity conditions. In other words, the differences between the initial states of the two weakly driven cavity modes vanish as time increases. Therefore, even in the chaotic regime, the two optical modes can still achieve a synchronized state.

Different from setup A1, the action of the common external drive in setup B1 is indirectly applied to the two weakly driven cavity modes via the mechanical coupling. The setup B1 highly relies on the mechanical coupling coefficient ${k}_{1}$ and ${k}_{2}$. The motion of the optical modes of weakly driven optomechanical systems is not only affected by its own oscillation but more crucially depends on the strongly driven optomechanical one. When the mechanical-coupling coefficients are large, setup B1 can be reduced to setup A1, and thus complete synchronization is achieved. However, for small ${k}_{1}$ and ${k}_{2}$, the motion of the mechanical modes is dominated by the weakly driven optical modes. Thus, the external drive has little effect on the optical cavity modes to be synchronized. As a result, in the weak mechanical-coupling regime, complete synchronization is impossible in setup B1.

### Phase synchronization

Phase synchronization is defined as the locking of the unwrapped phases in two dynamical systems. Below we will show phase synchronization of two chaotic optical modes in Fig. 2 in the strong-coupling small-detuning regime. Note that the unwrapped phases defined here are unfolded in every 2*π*-period. This is essentially different from the phases introduced for complete synchronization in Sec. II.

#### Phase synchronization in setup A2

**Figure 7:**
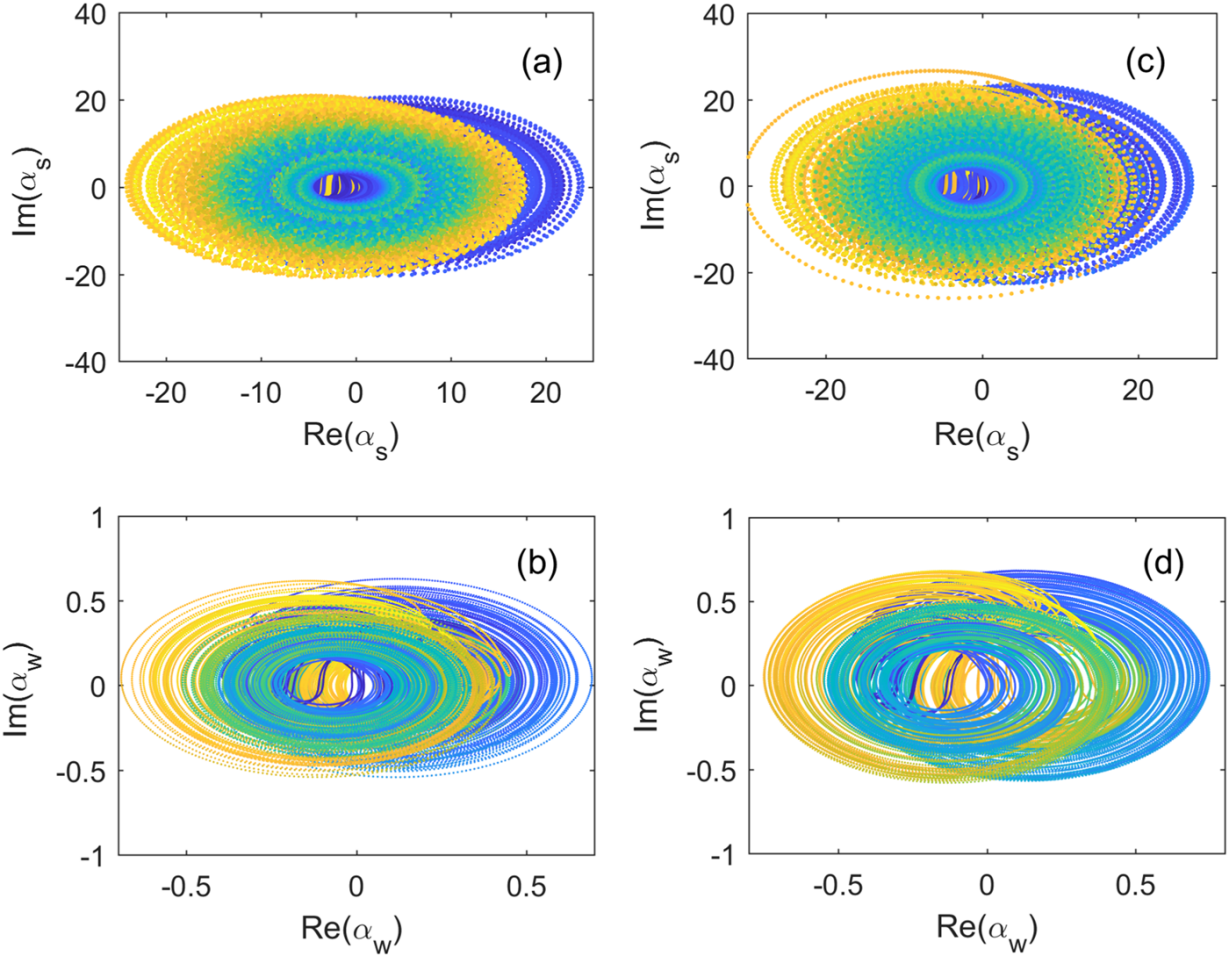
Phase synchronization for setups A2 and B2. Phase portraits of (**a**) the strongly driven and (**b**) weakly driven optomechanical systems in setup A2. While for setup B2, the corresponding phase portraits are shown in (**c** and **d**). The parameters for setup A2 are: ${\Delta }_{s}/2\pi =0.13\,{\rm{GHz}}$, ${\gamma }_{s}/2\pi =0.24\,{\rm{GHz}}$, ${g}_{s}/2\pi =0.126\,{\rm{GHz}}$, ${\varepsilon }_{s}/2\pi =15.4\,{\rm{GHz}}$, ${\Delta }_{w}/2\pi =26\,{\rm{MHz}}$, ${\gamma }_{w}/2\pi =52\,{\rm{MHz}}$, ${g}_{w}/2\pi =25.2\,{\rm{MHz}}$, ${\varepsilon }_{w}/2\pi =0.22\,{\rm{GHz}}$, ${\Gamma }_{m}/2\pi =2.8\,{\rm{MHz}}$, and ${\Omega }_{m}/2\pi =0.346\,{\rm{GHz}}$. The parameters for setup B2 are: ${\Gamma }_{s}/2\pi ={\Gamma }_{w}/2\pi =2.8\,{\rm{MHz}}$, ${\Omega }_{s}/2\pi ={\Omega }_{w}/2\pi =0.346\,{\rm{GHz}}$, and $k/2\pi =1.29\,{\rm{GHz}}$, while all the other parameters are the same as in setup A2.

In setup A2, shown in Fig. 2(a), the weakly and strongly driven optical modes are coupled via a mechanical mode. Here, the cavity mode ${\hat{a}}_{s}$ and mechanical mode $\hat{b}$ are prepared to chaotic motions when the cavity mode ${\hat{a}}_{s}$ is strongly driven. As a result, the weakly driven cavity mode is converted into a chaotic state via its coupling with the mechanical mode $\hat{b}$, which is also proved by the positive LLE. As shown in Fig. 7(a,b), the amplitudes of these two chaotic optical modes drastically change in different scales. However, two attractors rotate in the similar way with respect to the axis of ${\alpha }_{s}=0$ in Fig. 7(a) and ${\alpha }_{w}=0$ in Fig. 7(b), respectively. It indicates a correlation of the phases in the two attractors.

To study phase synchronization between the two chaotic optical modes, we calculate the ratio of the unwrapped phases of the strongly and weakly driven optical modes. To do so, we fix *G*_*s*_ but change the coupling strength *G*_*w*_ to see how the optomechanical coupling strength influences phase synchronization in the optomechanical system. The unwrapped phase ${\Psi }_{w}(t)$ [${\Psi }_{s}(t)$] of the weakly (strongly) driven cavity mode is evaluated from the real part of the observed signal ${\rm{Re}}[{\alpha }_{w}(t)]$ (${\rm{Re}}[{\alpha }_{s}(t)]$) with the analytic signal processing method. Phase synchronization occurs if the ratio of the phases of two nonidentical optical modes can be locked at a fixed value of ${G}_{s}/{G}_{w}$, as $t\to \infty$, according to our discussion in Sec. II.

**Figure 8:**
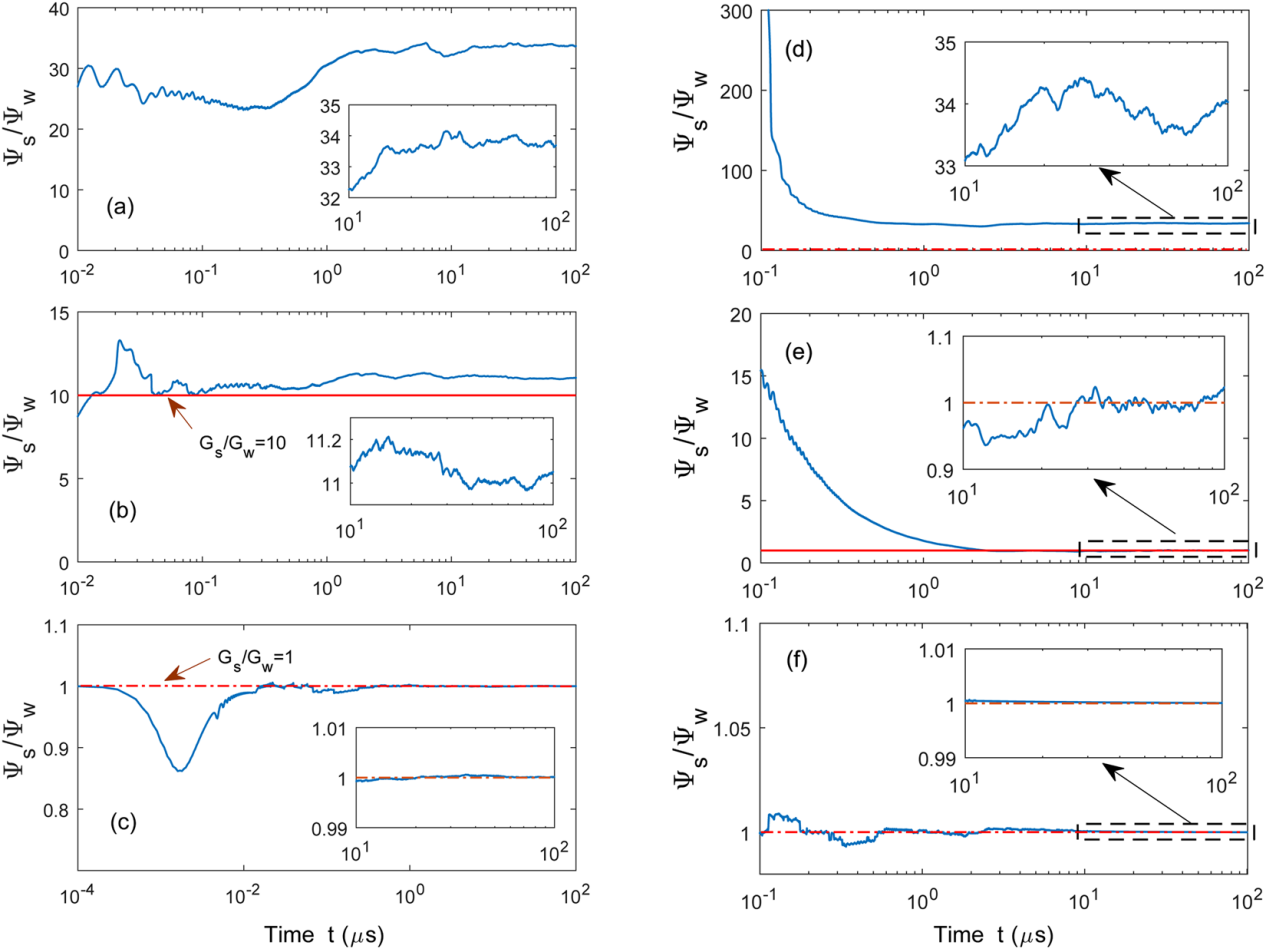
Phase synchronization for setups A2 [(**a**–**c**)] and B2 [(**d**–**f**)]: Evolutions of the ratios for the phases of the strongly [${\Psi }_{s}(t)$] and weakly [${\Psi }_{w}(t)$] driven cavity modes. In setup A2, we fix other parameters and change the coupling strengths to be: (**a**) ${G}_{s}/{G}_{w}=100$, (**b**) ${G}_{s}/{G}_{w}=10$, and (**c**) ${G}_{s}/{G}_{w}=1$, where ${G}_{s}={g}_{s}/{x}_{{\rm{ZPF}}}$ and ${g}_{s}=0.126\,{\rm{GHz}}$. In setup B2, the spring coefficients are: (**d**) $k/{\gamma }_{s}={10}^{-4}$, (**e**) $k/{\gamma }_{s}={10}^{-2}$, and (**f**) $k/{\gamma }_{s}={10}^{3}$, while ${g}_{s}/2\pi ={g}_{w}/2\pi =0.126\,{\rm{GHz}}$. The red line in each panel denotes the forecasting value ${G}_{s}/{G}_{w}$. Here, ${\varepsilon }_{w}/2\pi =1.1\,{\rm{GHz}}$ for setup A2 and ${\varepsilon }_{w}/2\pi =0.22\,{\rm{GHz}}$ for setup B2. All the other parameters are the same as in Fig. 7.

Figure 8(a–c) illustrate the evolutions of the ratio of unwrapped phase ${\Psi }_{s}(t)/{\Psi }_{w}(t)$ as a function of the coupling strength ${G}_{w}$. When ${G}_{w}$ is very weak, e.g. ${G}_{s}/{G}_{w}=100$, the motion of the weakly driven optical mode mainly depends on a given periodic input field. As a result, the ratio of ${\Psi }_{s}(t)/{\Psi }_{w}(t)$ fluctuates over a large region $[32,35.1]$ and does not converge, see Fig. 8(a). When ${G}_{w}$ is larger (e.g. ${G}_{s}/{G}_{w}=10$), the ratio of ${\Psi }_{s}(t)/{\Psi }_{w}(t)$ fluctuates within a relative smaller region, but still cannot approach to a constant value [see Fig. 8(b)] because the influence of the input field and the driving of the mechanical mode on the weakly driven optical mode compete with each other, leading to the randomly varying rhythms of the strongly and weakly driven cavity modes. In the strong-coupling regime, e.g. ${G}_{s}/{G}_{w}=1$, the phase of the weakly driven cavity mode is dominantly controlled by the chaotic mechanical mode. This mechanical mode also acts on the strongly driven cavity mode simultaneously. In this case, the resonance frequencies of both the weakly and strongly driven cavity modes are determined by the motion of the mechanical mode, see Fig. 8(c). The phase ratio converges to a constant value after oscillating over a transient period. These results show that phase synchronization can be realized in two chaotic optical oscillators, whereas their amplitudes are quite different. Moreover, the fixed value here is approximately equal to the ratio of optomechanical strengths ${G}_{s}/{G}_{w}=1$ [red dashed line in Fig. 8(c)], consistent with our theoretical analysis.

#### Phase synchronization in setup B2

In setup B2 shown in Fig. 2(b), the strongly and weakly driven optomechanical systems are coupled to each other with a rate $k$ via the mechanical coupling between two mechanical oscillators. When $k$ is strong enough, the setup B2 shares a similar dynamics with setup A2: the chaotic strongly driven optomechanical component (on the left-hand side) dominantly controls the motion of the weakly driven optomechanical component (on the right-hand) to a chaotic state, and their temporal phase can be locked at a constant value. As shown in Fig. 7, a typical chaotic attractor appears in the phase portrait [Fig. 7(d)] of the weakly driven optomechanical system when coupled to the chaotic strongly driven one [Fig. 7(c)].

As mentioned above, in phase synchronization, the unwrapped phases of the two chaotic optical cavity modes should be locked at the value ${G}_{s}/{G}_{w}$, which refers to the coupling strength of the strongly driven optomechanical part. To check the influence of $k$ on phase synchronization, we set ${G}_{s}/{G}_{w}=1$ and numerically calculate the temporal phases ${\Psi }_{w}(t)$ and ${\Psi }_{s}(t)$ for different mechanical-coupling strengths $k$ [see Fig. 8(d–f)]. Here, ${\Psi }_{w}(t)$ [${\Psi }_{s}(t)$] is obtained from the optical signals ${\rm{Re}}[{\alpha }_{w}(t)]$ (${\rm{Re}}[{\alpha }_{s}(t)]$) by Eq. (12) using the analytic signal processing method.

Similar to setup A2, the motion of the weakly driven optomechanical part is determined by two factors: (i) its inherent oscillation and (ii) the driving of the strongly driven optomechanical resonator. For the latter factor, the increase of the mechanical coupling coefficient $k$ enhances the coupling strength between the strongly and weakly driven components. When $k$ is very small ($k/{\gamma }_{s}={10}^{-4}$), see Fig. 8(d), the motion of the weakly driven optomechanical subsystem is separable from the strongly driven one. As a result, its motion is mainly determined by itself. Thus, the phases of the two cavity modes in two parts are uncorrelated. The phase ratio ${\Psi }_{s}(t)/{\Psi }_{w}(t)$ fluctuates in the range from 32 to 35 as the evolution time increases. As $k/{\gamma }_{s}$ increases to 10^−2^, the ratio ${\Psi }_{s}(t)/{\Psi }_{w}(t)$ oscillates around (but cannot stay at) the value ${G}_{s}/{G}_{w}=1$ as the evolution time progresses [see Fig. 8(e)]. In this case, the phase of the weakly driven optomechanical system is mainly dependent on its own oscillation and the external driving force. It can be seen in the inset of Fig. 8(e) that ${\Psi }_{s}(t)/{\Psi }_{w}(t)$ fluctuates in a much smaller range $[11,11.2]$, compared to the case in Fig. 8(d). When $k/{\gamma }_{s}={10}^{3}$, the motion of the weakly driven cavity mode is governed by the strongly driven optomechanical system. It leads to a perfect phase locking, as shown in Fig. 8(f). Note that there still exists a small discrepancy between ${\Psi }_{s}(t)/{\Psi }_{w}(t)$ and ${G}_{s}/{G}_{w}$, mainly because the temporal phases of the optical cavity modes are also effected by its own oscillation. This phase mismatch decreases as the mechanical coupling coefficient *k* increases.

#### Comparison of setups A2 and B2

Both setups A2 and B2 can be described as a common configuration in which the strongly driven optical mode dominates the motion of the weakly driven optical mode. To realize phase synchronization, setup A2 requires strong optomechanical coupling and weak detuning (the so-called strong-coupling small-detuning regime). Compared to setup A2, the setup B2 additionally requires a strong coupling between the two mechanical resonators. Moreover, in this strong mechanical coupling regime, the mathematic model of setup B2 is equivalent to that of setup A2.

## Conclusions and discussions

We have studied both complete and phase synchronization of optical cavity modes mediated by mechanical resonators. It is found that the complete synchronization of two identical optical cavity modes in chaotic motion can be obtained. We also showed the phase synchronization between two nonidentical optomechanical systems. In both types of chaotic synchronization, the chaotic displacement of the mechanical resonators is dominantly governed by the strongly driven optical mode. The chaotic motion of the mechanical resonators subsequently pulls the weakly driven optical cavity modes into chaotic motion. As a result, the phases of the strongly and weakly driven cavity modes can be synchronized. We stress again that complete synchronization can be achieved when the amplitude of the strongly driven cavity field is much larger than that of the weakly driven cavities: $|{\alpha }_{s}|\gg |{\alpha }_{1}|$ and $|{\alpha }_{s}|\gg |{\alpha }_{2}|$. While phase synchronization requires more conditions: ${G}_{s}\bar{x}\gg {\Delta }_{s}$ (${G}_{w}\bar{x}\gg {\Delta }_{w}$) and ${G}_{s}\bar{x}\gg {\gamma }_{s}$ (${G}_{w}\bar{x}\gg {\gamma }_{w}$). In this paper, the values of the strongly driven optomechanical resonators were based on the experimental data given in ref.^29^. The optomechanical models studied in this paper can be easily extended to a number of *N* chaotic optical modes, supporting either complete synchronization or phase synchronization, or both. Our work provides a method to observe chaotic synchronization in experimentally-accessible optomechanical systems. The setup of ref.^23^ can be used to experimentally implement our proposals. Future work can include the analysis of the synchronization of two chaotic mechanical resonators in optomechanical setups.
